# Self-powered ammonia synthesis under ambient conditions via N_2_ discharge driven by Tesla turbine triboelectric nanogenerators

**DOI:** 10.1038/s41378-020-00235-w

**Published:** 2021-01-18

**Authors:** Kai Han, Jianjun Luo, Jian Chen, Baodong Chen, Liang Xu, Yawei Feng, Wei Tang, Zhong Lin Wang

**Affiliations:** 1 CAS Center for Excellence in Nanoscience, Beijing Institute of Nanoenergy and Nanosystems, Chinese Academy of Sciences, Beijing, 100083 P.R. China; 2 School of Nanoscience and Technology, University of Chinese Academy of Sciences, Beijing, 100049 P.R. China; 3 Center on Nanoenergy Research, School of Physical Science and Technology, Guangxi University, Nanning, 530004 P.R. China; 4 School of Material Science and Engineering, Georgia Institute of Technology, Atlanta, GA 30332-0245 USA

**Keywords:** Environmental, health and safety issues, Chemistry

## Abstract

Ammonia synthesis using low-power consumption and eco-friendly methods has attracted increasing attention. Here, based on the Tesla turbine triboelectric nanogenerator (TENG), we designed a simple and effective self-powered ammonia synthesis system by N_2_ discharge. Under the driving of the simulated waste gas, the Tesla turbine TENG showed high rotation speed and high output. In addition, the performance of two Tesla turbine TENGs with different gas path connections was systematically investigated and discussed. A controllable series-parallel connection with the control of gas supply time was also proposed. Taking advantage of the intrinsic high voltage, corona discharge in a N_2_ atmosphere was simply realized by a Tesla turbine TENG. With the flow of N_2_, the generated high-energy plasma can immediately react with water molecules to directly produce ammonia. The self-powered system achieved a yield of 2.14 μg h^−1^ (0.126 μmol h^−1^) under ambient conditions, showing great potential for large-scale synthesis.

## Introduction

Developing novel energy-saving and eco-friendly methods and techniques to synthesize substances and materials is receiving increasing attention. Ammonia is an important inorganic chemical product, and its classical Haber–Bosch synthetic process requires harsh conditions, such as high temperatures and high pressures, which have complex adverse effects on energy and the environment^1,2^. Since the triple bond of N_2_ is extremely stable, various attempts and efforts have been made to break it under mild conditions, such as biological catalytic^3^, electrocatalytic^4–8^, photocatalytic^8,9^, and discharging methods^10–13^. However, the selection processes of specialized catalysts are still significant challenges^14–16^. By contrast, the discharge method more easily breaks nitrogen–nitrogen bonds due to its simple device structure^10–13^. Since high voltage is an essential condition for this process, it is necessary to select an appropriate technology from the perspective of energy and environment.

Recently, triboelectric nanogenerators (TENGs), a technology in energy harvesting, have been developing at a rapid pace^17–19^. Originating from Maxwell’s displacement current, TENGs have been utilized to convert multiform mechanical energy into electric energy for a wide variety of applications in different fields^20–31^. Taking advantage of the intrinsic property of high voltage, a strong electric field can be easily built to break chemical bonds, which is convenient for N_2_ fixation^32,33^. Moreover, with the help of an appropriate configuration of the TENG, the discharge process can become self-powered. As previously reported, the residual kinetic energy of waste gas can be used as a source of mechanical energy to drive the TENG for self-powered synthesis^32^. However, this application is inefficient as a primary use of waste gas. Thus, further research on better energy management of the gas flow will be favorable in practical applications.

In this work, a simple and efficient self-powered ammonia synthesis system via N_2_ discharge was designed and fabricated. A Tesla turbine device was introduced to achieve a higher mechanical energy conversion efficiency^34–39^. By using the bladeless turbine, the gas kinetic energy could be transformed into high-speed rotation energy to drive the TENG for power generation. In addition, the performance of two Tesla turbine TENGs in different gas-line connections was studied. Driven by the simulated waste gas, N_2_ discharge can be simply achieved by utilizing the intrinsic high voltage of the TENG. Moreover, using water as the hydrogen source, the generated high-energy plasma will directly react with the water molecules as the N_2_ flows through. Then, ammonia will be formed as a product of the reaction in the self-powered system.

## Results and discussion

### The Tesla turbine TENG and ammonia synthesis mechanism

**Fig. 1:**
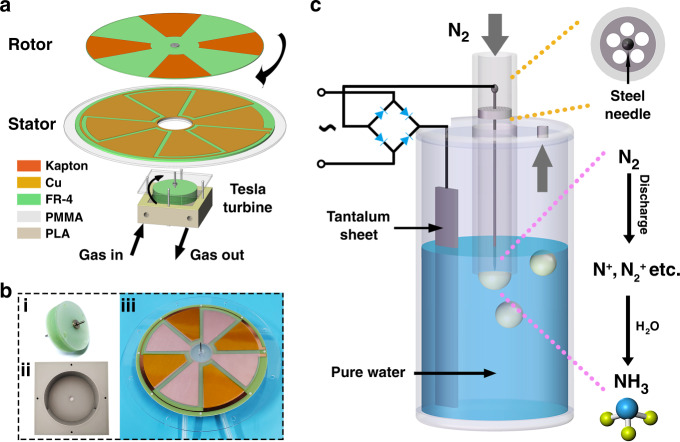
Schematic diagrams and photographs. **a** Schematic diagram of the Tesla turbine TENG. **b** Photographs of (i) the bladeless turbine with the driving shaft, (ii) the 3D printing casing, and (iii) the disk TENG. **c** Schematic diagram of the ammonia synthesis device and main reaction processes.

As shown in Fig. 1a, the Tesla turbine TENG is an integration of the Tesla turbine driving device and the disk TENG. The former is composed of a three-dimensional (3D) printing casing and a bladeless turbine, whose working principle is based on the boundary layer effect of the fluid. Influenced by the viscous force, a thin boundary layer will be formed on the edge of the object. The velocity increases with increasing distance from the boundary layer. By utilizing this effect, a group of disks called bladeless turbines can be rotated at high speeds by a fast-moving fluid such as gas. For optimal gas flow management and further kinetic energy utilization, an inlet and an outlet were symmetrically designed on the same side of the casing. The cutaway view of the casing is shown in Fig. S1a, in which a clear inner gas flow channel can be seen. The bladeless turbine is made up of dozens of epoxy fiberglass (FR-4) disks with both a thickness and space distance of 0.2 mm (Fig. S1b). For the disk TENG, a noncontact-sliding freestanding mode was chosen to achieve high speed for high output^32^. The working principle of the TENG is shown in Fig. S2. To obtain a higher intrinsic voltage, the number of precharged Kapton layers on the rotor was set at four for a larger single area of ~33 cm^2^. Correspondingly, there were four pairs of electrodes on the printed circuit board (PCB) stator, which was fixed on an acrylic substrate. The detailed assembly process of the Tesla turbine TENG is shown in Fig. S3 and described in the materials and methods section. Figure 1b shows photographs of the bladeless turbine, the 3D printing casing, and the disk TENG. As the gas flow passes through the Tesla turbine driving device, the turbine starts to run, driving rotor movement and generating electric energy.

A schematic diagram of the ammonia synthesis device and main reaction processes is shown in Fig. 1c. A steel needle is fixed in the center of an acrylic tube as the negative electrode for point discharge. To ensure N_2_ passes through, several holes are reserved in the fixation part. The needle point is level with the section of the tube, and both are submerged in pure water. When N_2_ is at the inlet, many bubbles will be formed around the end of the tube. Since the other electrode is connected with the positive pole of the rectified TENG, a good oxidation-resistant and relatively inexpensive material, tantalum (Ta), was chosen. After rectification, the high voltage produced by the TENG is utilized to generate a strong electric field in the space between the tip and the surface of the bubble, i.e., the water layer, for N_2_ discharge. Under an electric field, nitrogen molecules will be excited and ionized, leading to the breakage of the nitrogen–nitrogen triple bond and forming a series of high-energy ions such as N^+^ and N_2_^+^^10,11^. Then, with the flow of N_2_, these ions will directly react with water molecules in a short time. Herein, the role of water is not only as an essential part of building the electric field but also as the reactant. A possible reaction mechanism is proposed and simply described below. Under the driving of the TENG, nitrogen ions will further obtain electrons, and some nitrogen atoms may go into a low valence state to form soluble ionic state ammonia by combining with hydrogen from water. Moreover, the oxygen atoms in the water are likely to be oxidized into O_2_ at the anode region.

### Basic performance

**Fig. 2:**
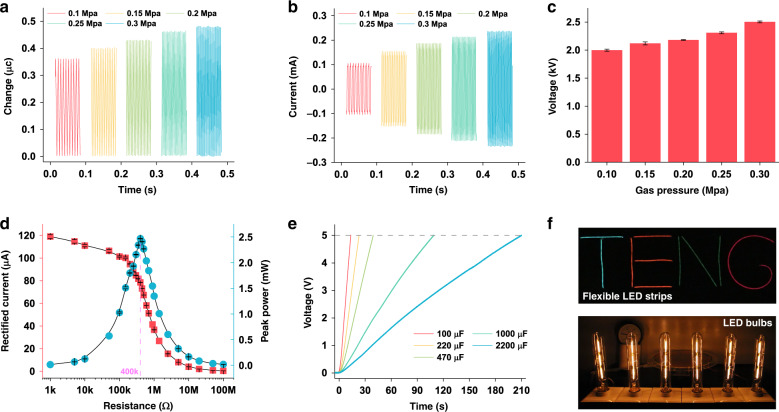
Basic performance of the Tesla turbine TENG. **a** Transferred charge. **b** Short-circuit current. **c** Open-circuit voltage. **d** Rectified current and peak power with different external resistances at gas pressures of 0.12–0.13 MPa. **e** Charging performance with different capacitors at gas pressures of 0.12–0.13 MPa. **f** Video captures of lighting the flexible LED strips and the LED bulbs at gas pressures of ~0.18–0.2 MPa.

Figure 2 shows the basic performance of the Tesla turbine TENG. A high-pressure N_2_ bottle and an air compressor were used as the gas sources, supplying different gas pressures and long-term drive. With increasing gas pressure from 0.1 to 0.3 MPa, the transferred charge increases gradually from 0.36 to 0.49 μC (Fig. 2a). The correspondence between rotation speed and gas pressure was also investigated, as shown in Fig. S4. The rotation speed increases from over 2000 r min^−1^ to almost 5000 r min^−1^. As previously reported, the FR-4 substrate of the rotor is prone to contact Cu electrodes, generating a positive charge at a low rotation speed due to its ultrathin structure, which will further reduce the transferred charge^32^. The variation in transferred charge after 1 h of continuous working indicates this negative effect as well (Fig. S5). Figure 2b shows the short-circuit current results; ~0.24 mA is reached under a pressure of 0.3 MPa. In addition, the TENG exhibits high open-circuit voltage properties with a range of 2–2.5 kV for potential gas discharge applications (Fig. 2c). Driven by an air compressor at a gas pressure of 0.12–0.13 MPa, the rectified current and the peak power with different external resistances are shown in Fig. 2d. The maximum peak power reaches 2.5 mW with a matching resistance of 400 kΩ at a rotation speed of ~2600 r min^−1^. The charging performance was tested under the same driving conditions (Fig. 2e). It takes ~10 s to charge a 100 μF capacitor to a common electronic device working voltage of 5 V. Even for a larger capacity up to 2200 μF, the charging time is <4 min. Ten flexible, colored LED strips with a total length of almost 2.4 m and six LED bulbs were successfully lit by the Tesla turbine TENG, further demonstrating its excellent output performance (Fig. 2f, Supplementary Videos 1 and 2), with a gas pressure of ~0.18–0.2 MPa. For the flexible strips, a voltage booster circuit and a needle-to-needle-point device were used to achieve a higher voltage for high instantaneous current by air discharging in pulse mode (Fig. S6). During the discharging process, the air is ionized into plasma, and plenty of free charges are released to form a conductive channel for effective electricity collection^40–42^. When lighting the LED bulbs, no extra device was involved. Overall, the energy conversion efficiency is still low. An estimated calculation is shown in the Supplementary Information. However, when the rotor rotates at a steady state, it theoretically only needs to overcome the electrostatic force to work for electricity. A large amount of energy is lost in the process of gas transportation and mechanical energy conversion due to various unwanted frictions. In addition, there is still much residual energy in the output gas.

### Performance in different connection modes

**Fig. 3:**
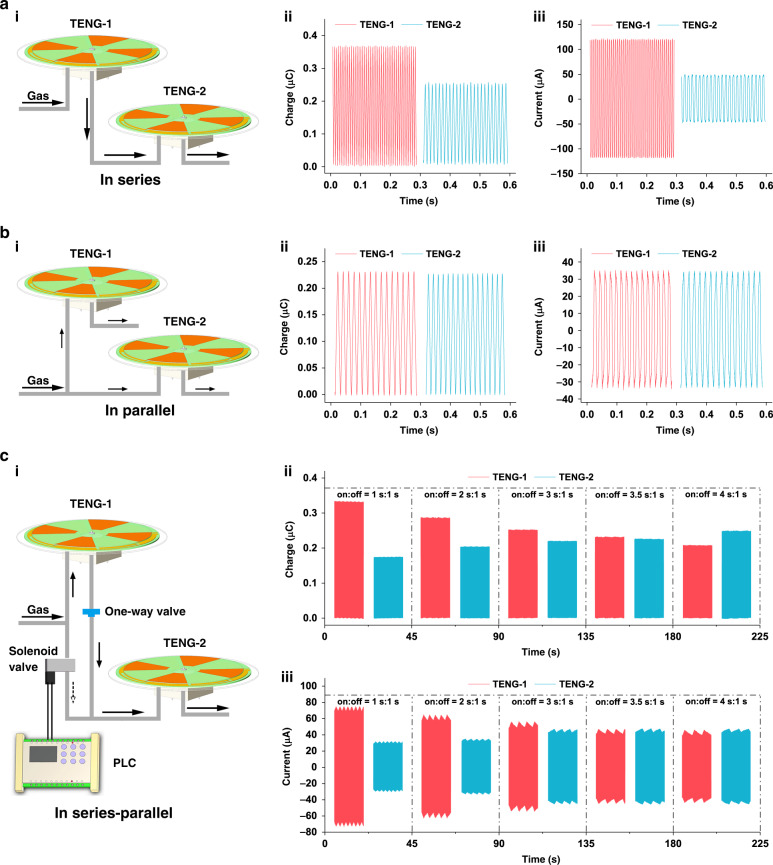
Performance in different connection modes. Performance of two Tesla turbine TENGs in **a** series connection, **b** parallel connection, and **c** series-parallel connection modes. (i) Schematic diagram. (ii) Transferred charge. (iii) Short-circuit current.

To better harness the kinetic energy of the gas flow, the performance of two Tesla turbine TENGs (named TENG-1 and TENG-2) in different gas path connections was investigated. Since additional electrons involved in the reaction would be better for the synthesis, transferred charge and short-circuit current are two key parameters for evaluating the energy harvesting efficiency of the TENG. Figure 3a shows the testing results under the serial connection mode. Compared with that when only one TENG was driven (Fig. S7), the performance of TENG-1 was not significantly different, while the performance of TENG-2 decreased. The degradation is attributed to the kinetic energy consumption of TENG-1. Such a performance difference will lead to uneven yields, bringing about the load balance problem in the product line. For the parallel connection (Fig. 3b), the performance of the two TENGs is very similar. However, both the transferred charge and short-circuit current are much lower than those of a single TENG. It is inferred that the diversion of gas flow leads to less kinetic energy in each channel, which is not conducive to the efficient conversion for the Tesla turbine device^39^. In addition, the results of rotation speed (Fig. S8) and open-circuit voltage (Fig. S9a, b) show a similar trend in these two modes.

To adjust the two TENGs’ performance in the flow line, we proposed a controllable series-parallel connection mode. As shown in Fig. S10, when stopping the gas supply, a Tesla turbine TENG can keep rotating and outputting for several seconds under the effect of rotational inertia. Thus, a solenoid valve with a programmable logic controller (PLC) and a one-way valve were used in the gas path (Fig. 3c(i)) to control the gas supply time and prevent the backwardness of gas when TENG-2 was supplied, respectively. When the solenoid valve is off, the gas path is in a simple series connection. When the solenoid valve is on, the gas path is a hybrid connection. TENG-2 can obtain the residual energy of the TENG-1 path and the kinetic energy of its own gas path. To avoid reducing the rotation speed of TENG-1 too much, an off time of 1 s was chosen as a fixed parameter. With the control of the PLC, a series of tests at different gas supply times were conducted. As illustrated in Fig. 3c(ii) and (iii), the performance of TENG-1 gradually decreases with increasing gas supply time for TENG-2. Correspondingly, the performance of TENG-2 increased. At the on-off time ratio of 3.5 s:1 s, a similar output appears. Compared with the direct parallel connection, although the amount of transferred charge does not change significantly, the current increases by ~20–38%. The results of the open-circuit voltage also validate the effectiveness of this connection mode (Fig. S9c). When compared with series connections, there is a certain decrease in output performance. However, both TENGs can be easily adjusted into a relatively uniform state for better management in the synthetic process.

### Self-powered ammonia synthesis

**Fig. 4:**
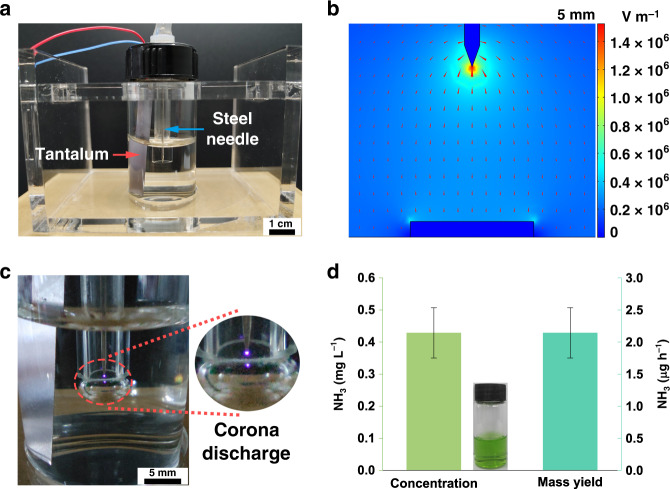
Self-powered ammonia synthesis system. **a** Photograph of the ammonia synthesis device. **b** Simulation image of the N_2_ discharge with the simplified model. **c** Photograph of the N_2_ corona discharge. **d** Concentration and mass yield of ammonia after 2 h of self-powered synthesis.

To achieve N_2_ discharge and better use of high-energy ions, a simple ammonia synthesis device was designed. The paragraph of the device is shown in Fig. 4a. As described above, the Tesla turbine TENG can provide a high voltage up to over 2 kV. Based on this, we made a COMSOL simulation of N_2_ discharge. For the purpose of simplicity, a needle-plate model is used to simulate the discharge state in the N_2_ atmosphere. The materials of the needle and the bottom electrode were set as steel and water, respectively. For the physical field, the corona discharge model was chosen. Taking the needle as the reference, the voltage between two electrodes was set at −2 kV. A series of simulation results with different distances from the tip to the plate are illustrated in Fig. S11. Figure 4b is a partially enlarged image at the distance condition of 5 mm. It is worth noting that a high electric field over 10^6^ V m^−1^ can be easily obtained at the tip to cause the ionization of N_2_. Figure 4c is a photograph of the N_2_ discharge, which is driven by the Tesla turbine TENG under a gas pressure of 0.12–0.13 MPa. The corona discharge can be clearly seen at the tip of the needle. To reduce the interference of potential substances from the N_2_ source itself, a control experiment was conducted without the driving of the Tesla turbine TENG. After ventilation for 6 h, no ammonia was detected within the detection limit (Fig. S12), indicating that the gas-washing devices could commendably eliminate adverse effects. Figure 4d shows the determination results after 2 h of self-powered synthesis. Ammonia was successfully synthesized. By calculation, the yield of ammonia can reach 2.14 μg h^−1^ (0.126 μmol h^−1^). To eliminate unknown negative effects from the use of Ta sheets, another synthetic experiment using platinum (Pt) sheets as electrodes in water was conducted, as shown in Fig. S13, further indicating the feasibility and reliability of our TENG-based self-powered synthetic method and the potential for further large-scale application (Fig. S14).

### Conclusion

In summary, by utilizing the high voltage generated from the Tesla turbine TENG, a self-powered ammonia synthesis system was demonstrated. Under ambient conditions, high-energy plasma produced from N_2_ discharge can directly react with water, forming ammonia products. A yield of 2.14 μg h^−1^ (0.126 μmol h^−1^) was achieved at a simulated waste gas pressure of 0.12–0.13 MPa provided by an air compressor. To improve the utilization efficiency of the gas kinetic energy, the performance of two Tesla turbine TENGs in different gas path connections was systematically investigated. A controllable series-parallel connection mode was proposed. By controlling the gas supply time, the performance of each TENG was effectively improved compared with parallel connections and showed better proximity than in series connections. Based on the above, such a fast and straightforward method for ammonia synthesis shows great potential to achieve large-scale synthesis applications.

## Materials and methods

### Fabrication of the Tesla turbine TENG

The size of the 3D printing casing was 7 cm × 7 cm × 2 cm, and the diameter of the central aperture was 5.2 cm. One bearing hole and four screw holes were reserved at the bottom and around the framework of the casing. The thickness of the whole bladeless turbine was ~1.5 cm. The diameter and thickness of each FR-4 disk were 5 cm and 0.2 mm, respectively. The connections between the disks were achieved by four small pieces of the same FR-4 with a size of ~3 mm × 3 mm and instant adhesive. For the disk TENG, the rotor substrate was an FR-4 disk with a diameter of 20 cm and a thickness of 0.3 mm to balance weight and strength. At the center, a hole was reserved for the connection with the driving shaft. Four pieces of 60-μm-thick fan-shaped Kapton film were stuck on the FR-4 substrate. Then, the Kapton films were fully precharged by friction with Cu foil in advance. The charge density (2.13 × 10^−4^ C m^−2^) was estimated by testing the average surface electrostatic potential (Fig. S15). More details are shown in the Supplementary Information. The area of each Kapton film was ~33 cm^2^. The stator was a custom-made PCB with four pairs of Cu electrodes on a polymethyl methacrylate (PMMA) substrate. As shown in Fig. S3, a high-speed bearing was first installed in the casing, followed by a matched driving shaft. Then, the bladeless turbine was fixed with the shaft. A silicone gasket was added above the casing to prevent gas flow leakage. After the PMMA plate with a bearing was installed, the Tesla turbine device was completed. A larger PMMA substrate was then added for the fixation of the PCB board. Finally, the rotor was fixed to the driving shaft by two 1-mm-thick PMMA pieces with a diameter of 1 cm. A small vertical distance (<0.5 mm) was set between the rotor and the bottom electrode. In addition, two metallic gaskets were stuck at two ends of the bladeless turbine to reduce potential friction in our experiments.

### Measurements of the basic performance of the Tesla turbine TENG

A high-pressure N_2_ bottle (99.999%, Praxair) and an air compressor (2 × 1500 W, 280 L min^−1^, Ortus) were selected as the driving sources. An electrometer (6514, Keithley) and a multimeter with a high-voltage attenuation rod (HVP-40, Pintech) were used to measure the performance. In addition, a gas pressure gauge was added to the main as line to monitor the pressure condition above the atmospheric pressure. During the peak power and charging performance tests, the air compressor was adjusted to a stable pressure supply state of 0.12–0.13 MPa. For testing the ability of the apparatus to light flexible, colored LED strips (LS-D03, 7.36 W m^−1^, 12 V DC, Blue Shark) and LED bulbs (3 W, 220 V AC, Designer Lamp), a gas pressure of 0.18–0.2 MPa, which was slightly higher at the primary working state of the air compressor due to the stored gas in the gas tank, was applied.

### Measurements of the performance in different gas path connections

Two Tesla turbine TENGs with the same structure were connected in series, parallel, and controllable series-parallel modes. The first two connection modes were simply realized by linking flexible gas tubes of the same size. For the controllable series-parallel connection, the inlet of TENG-1 was connected with one branch of the main gas path, and the outlet was connected with a one-way valve. The inlet of TENG-2 was connected with the one-way valve and a solenoid valve (2W-200-20, Trilobite) in another branch of the main gas path. The solenoid valve was controlled by a PLC (PLC-wifi, Shuangyuan) device. The performance of two TENGs was measured by the same electrometer and the multimeter with a high-voltage attenuation rod.

### Fabrication of the ammonia synthesis device

A normal steel needle was fixed in the middle of a small acrylic board with holes. Then, both the board and needle were set in the center of an acrylic tube with an inside diameter of 4 mm and an outside diameter of 6 mm. The acrylic tube was fixed on the reaction bottle cap, and one end was connected with the nitrogen source. An N_2_ outlet hole was also reserved in the cap. The size of the tantalum sheet was 2.5 cm × 0.7 cm × 0.1 mm.

### Self-powered ammonia synthesis

The N_2_ (99.999%, Praxair) flow rate was set at 0.15 L min^−1^. A total of 40 mL of acid solution (H_2_SO_4_, 0.05 mol L^−1^) and 40 mL of alkali solution (NaOH, 0.1 mol L^−1^) were utilized to wash N_2_ before it passed through the reaction container. The control test with only N_2_ passing through 10 mL water for 6 h was repeated three times. In a typical synthesis process, 12 mL of pure water was first added into the reaction container. Then, 2 mL of water was removed as the initial blank control. After 2 h of self-powered synthesis driven by the Tesla turbine TENG at gas pressures of 0.12–0.13 MPa, 2 mL of solution was taken for detection. The standard calibration curve of ammonia is shown in Fig. S16. To ensure a stable output, the Kapton films were charged by friction with Cu every 30 min during the synthetic process.

## Supplementary information

Supplementary Information

supporting video 1

supporting video 2
