# Supplementary material for: Self-powered ammonia synthesis under ambient conditions via N2 discharge driven by Tesla turbine triboelectric nanogenerators
Source: Microsyst Nanoeng. 2021 Jan 18;7:7. doi: 10.1038/s41378-020-00235-w (PMC8433223; doi:10.1038/s41378-020-00235-w)
Supplement: Supplementary file 1 — Supplementary Information [file 41378_2020_235_MOESM1_ESM.docx]

**Supplementary Information**

**Self-powered ammonia synthesis under ambient conditions via N_2_ discharge as driven by Tesla turbine triboelectric nanogenerators**

Kai Han^1,2^, Jianjun Luo^1,2^, Jian Chen^1,2^, Baodong Chen^1,2^, Liang Xu^1,2^, Yawei Feng^1,2^, Wei Tang^*,1,2,3^ and Zhong Lin Wang^*,1,2,3,4^

1 CAS Center for Excellence in Nanoscience, Beijing Institute of Nanoenergy and Nanosystems, Chinese Academy of Sciences, Beijing 100083, P. R. China

2 School of Nanoscience and Technology, University of Chinese Academy of Sciences, Beijing 100049, P. R. China

3 Center on Nanoenergy Research, School of Physical Science and Technology, Guangxi University, Nanning 530004, P. R. China

4 School of Material Science and Engineering, Georgia Institute of Technology, Atlanta, Georgia 30332-0245, USA

**Chemical Reagents**

Concentrated sulfuric acid (Beijing Chemical Works, H_2_SO_4_, 98%), sodium hydroxide (Beijing Chemical Works, NaOH, AR, 99%), salicylic acid (Aladdin, C_7_H_6_O_3_, AR, 99.5%), sodium citrate dihydrate (MACKULIN, Na_3_C_6_H_5_O_7_·2H_2_O, AR, 99.0%), ammonium nitrate-15N (Aladdin, NH_4_^15^NO_3_, 99atom%, ≥98.5%), sodium hypochlorite solution (Aladdin, NaClO, available chlorine, ≥5.0%), sodium nitroferricyanide dihydrate (Aladdin, C_5_FeN_6_Na_2_O·2H_2_O, 99.98% metals basis).

**Determination of NH_3_**

The indophenol blue method is used. 2 mL of solution is removed from the reaction container as the test sample. 2 mL of mixed detection solution (1 mol L^-1^ NaOH, 5 wt% C_7_H_6_O_3_ and 5 wt% Na_3_C_6_H_5_O_7_) is first added into the sample, then 1 mL of 0.05 mol L^-1^ NaClO and 0.2 mL of 1 wt% C_5_FeN_6_Na_2_O were added in turn. After 2 h, the sample is measured by an ultraviolet-visible spectrophotometer (UV-3600, SHIMADZU Ltd.) at the characteristic absorption wavelength of 655 nm .


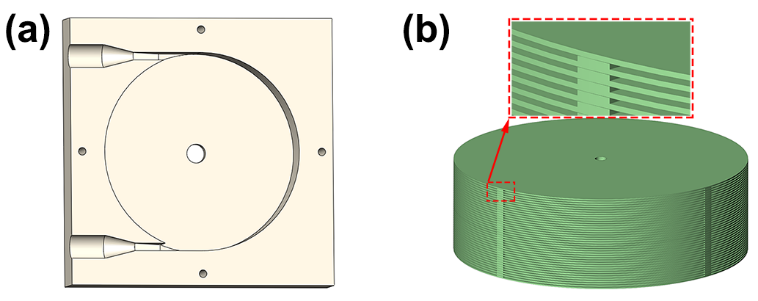


**Fig. S1** Schematic diagram of (a) the cutaway view of the 3D casing and (b) the bladeless turbine.


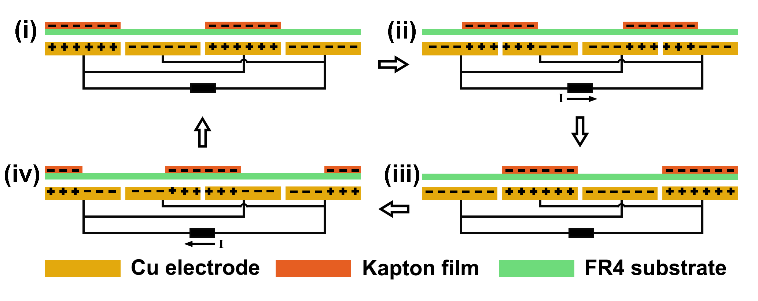


**Fig. S2** Working princple of the non-contact-sliding freestanding mode TENG.


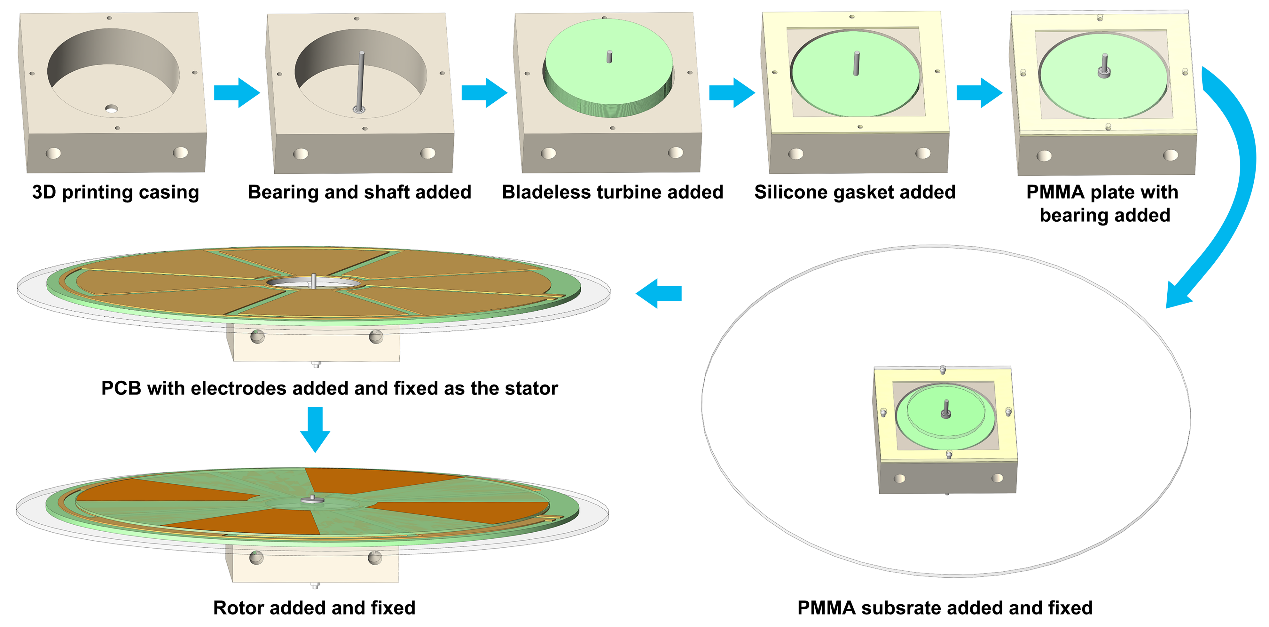


**Fig. S3** Assembling process of the Tesla turbine TENG.





**Fig. S4** Corresponding relation between rotation speed and gas pressure.


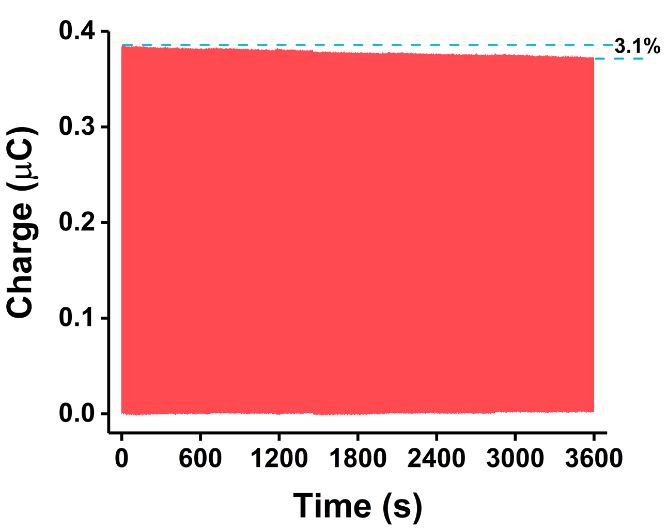


**Fig. S5** Variation of transferred charge in 1 h continuous working.


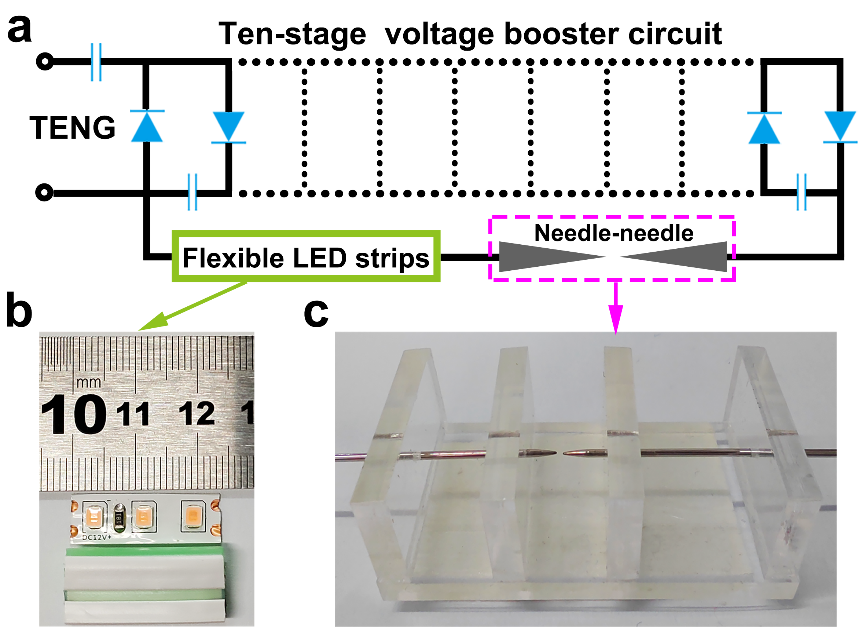


**Fig. S6** (a) Schematic diagram of the ten stage voltage booster circuit. (b) Photograph of a typical section of the flexible LED strips. (c) Photograph of the needle-to-needle-point device.

**An estimated energy conversion efficiency**

The flow energy was transformed into the rotational energy of the bladeless turbine (m1=28.5g, r1=2.5 cm) and the rotor (m1=17.7g, r1=10 cm). By using the rotational kinetic energy formula below, the total energy acquired is about 3.61 J at the speed of 2600 r min^-1^.

$E_{k}=\frac{1}{2}J\omega^{2}$; $J=\frac{1}{2}mr^{2}$

The instantaneous driving power for electricity generation is 3.61 W. As shown in Figure 2d, the maximum peak power by one TENG is 2.5 mW with a matching resistance of 400 kΩ. The overall efficiency is about 0.07%.


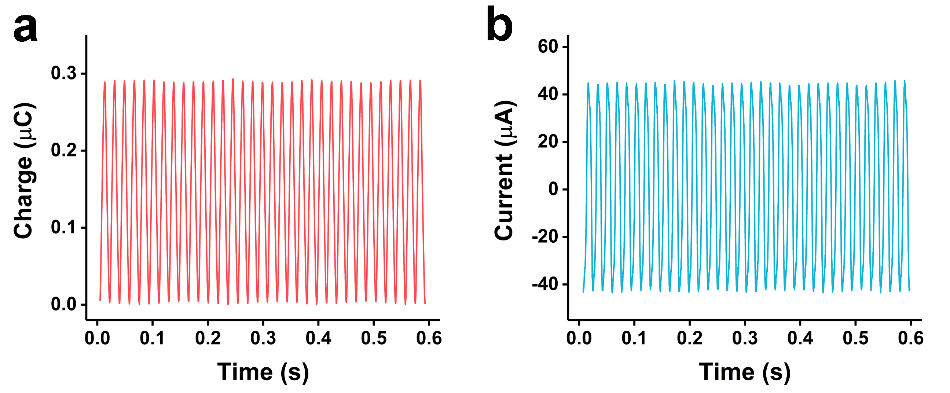


**Fig. S7** Performance of a single Tesla turbine TENG under the gas pressure of 0.12-0.13 MPa. (a) Transferred charge, (b) Short-circuit current.


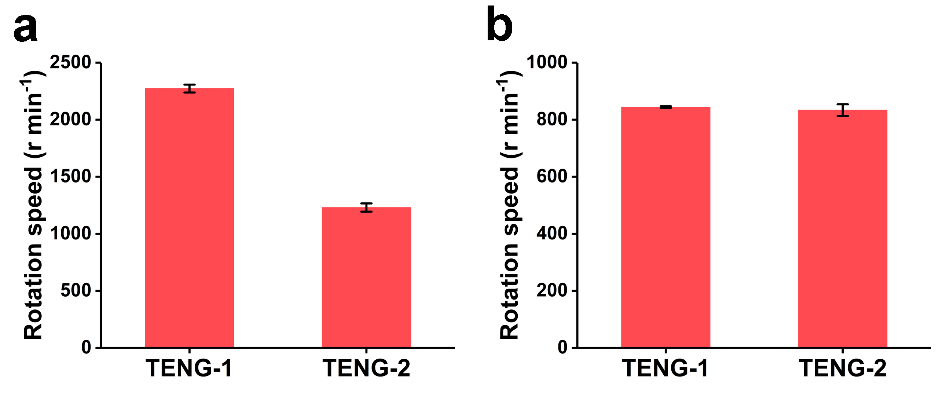


**Fig. S8** Rotation speeds of two Tesla turbine TENGs (a) in series connection, (b) in parallel connection.


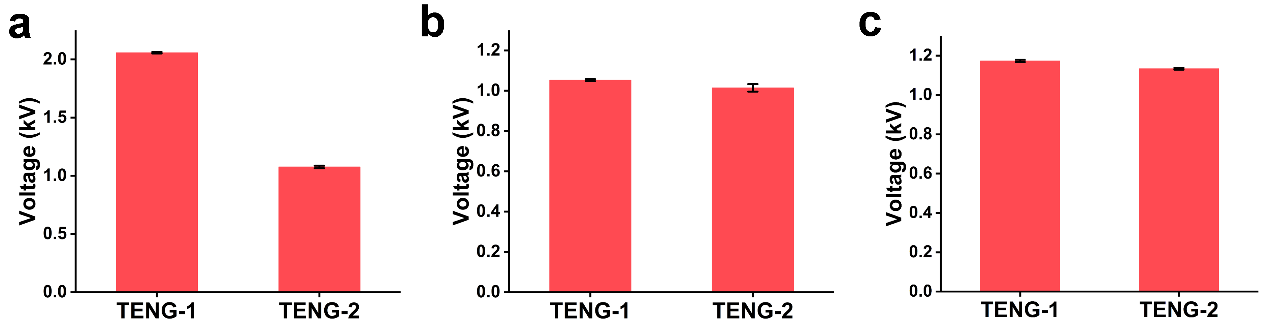


**Fig. S9** Open-circuit voltage of two Tesla turbine TENGs (a) in series connection, (b) in parallel connection and (c) in series-parallel connection.


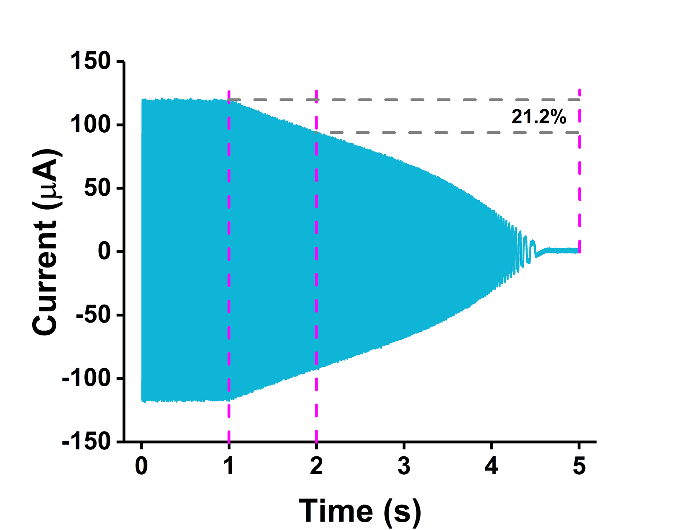


**Fig. S****10** Current attenuation curve of a single Tesla turbine TENG when the supply gas was stopped.


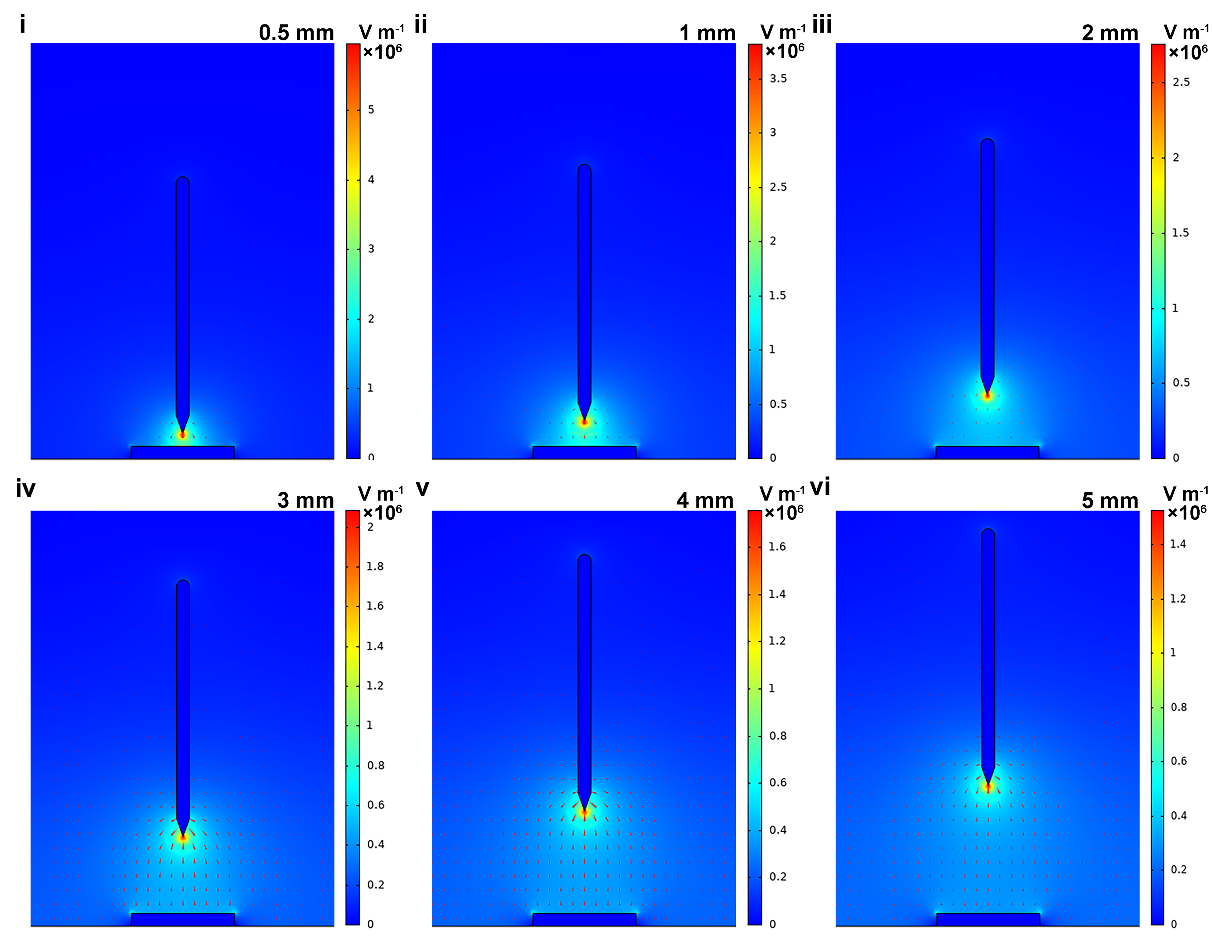


**Fig. S11** COMSOL simulation results of N_2_ discharge with different distances from the tip to the plate.


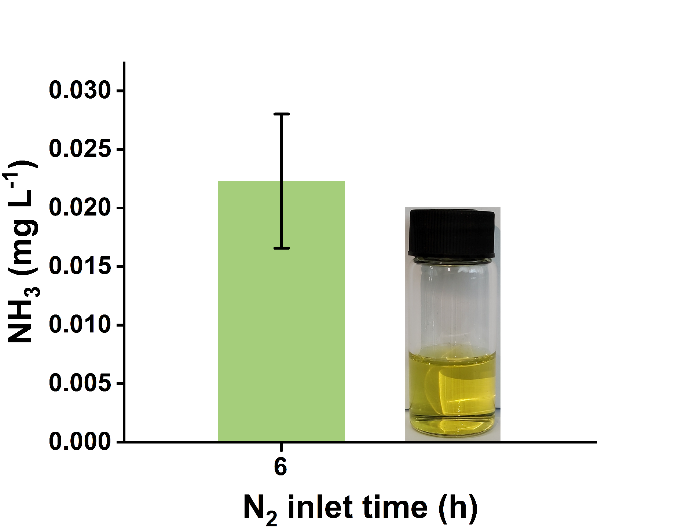


**Fig. S12** Control test without the drive of the Tesla turbine TENG for 6 h N_2_ ventilation.


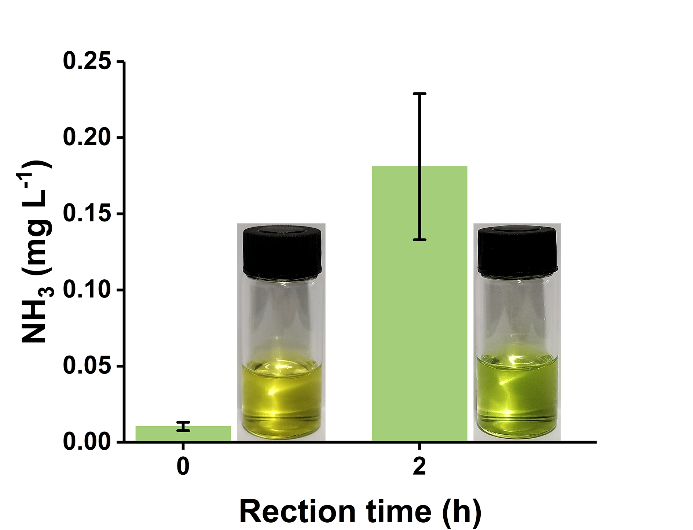


**Fig. S13** Concentration yield of ammonia after 2 h self-powered synthesis using Pt sheet as the electrode.


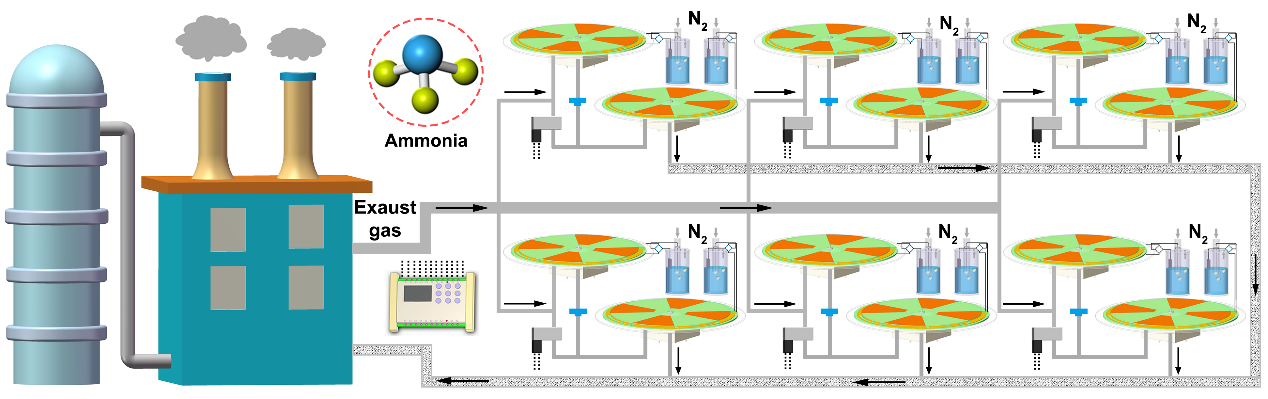


**Fig. S14** Schematic diagram of the large-scale strategy for ammonia synthesis.


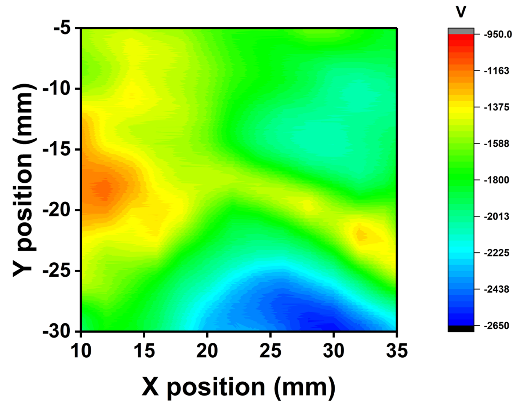


**Fig. S15** Surface electrostatic potential distribution of the pre-charged Kapton film (2.5×2.5 cm).

A regular shape of the pre-charged Kapton film was tested by an electrostatic voltmeter (Trek, Model 347). The average surface electrostatic potential is 1746.6 V. The value of charge density σ is estimated according to the expression.

$$\sigma=\frac{\varepsilon_{0}\varepsilon_{r}V}{d}$$

*ε_0_*=8.85×10^-12^ F m^-1^, *ε_r1_*=3.5, *d_1_*=60 μm, *ε_r2_*=5.4, *d_2_*=0.3 mm, *d=d_1_+d_2_*, *V*=1746.6 V. Since the FR-4 substrate is also put in the testing system, the equivalent dielectric constant (*ε_r_*) is 4.95 by using the series formula of capacitance.

$$C=\frac{\varepsilon_{0}\varepsilon_{r}S}{d}$$

$$\frac{1}{C}=\frac{1}{C_{1}}+\frac{1}{C_{2}}$$

**

**

**Fig. S16** Standard calibration curve of different concentrations of NH_3_.
